# Enzymatic Antioxidant Signatures in Hyperthermophilic Archaea

**DOI:** 10.3390/antiox9080703

**Published:** 2020-08-03

**Authors:** Emilia Pedone, Gabriella Fiorentino, Simonetta Bartolucci, Danila Limauro

**Affiliations:** 1 Istituto di Biostrutture e Bioimmagini, CNR, Via Mezzocannone 16, 80134 Napoli, Italy; empedone@unina.it; 2 Dipartimento di Biologia, Università degli Studi di Napoli Federico II, Complesso universitario Monte S. Angelo, Via Cinthia, 80126 Napoli, Italy; fiogabri@unina.it (G.F.); bartoluc@unina.it (S.B.)

**Keywords:** antioxidant enzymes, oxidative stress, reactive oxygen species, hyperthermophiles, archaea

## Abstract

To fight reactive oxygen species (ROS) produced by both the metabolism and strongly oxidative habitats, hyperthermophilic archaea are equipped with an array of antioxidant enzymes whose role is to protect the biological macromolecules from oxidative damage. The most common ROS, such as superoxide radical (O_2_^•−^) and hydrogen peroxide (H_2_O_2_), are scavenged by superoxide dismutase, peroxiredoxins, and catalase. These enzymes, together with thioredoxin, protein disulfide oxidoreductase, and thioredoxin reductase, which are involved in redox homeostasis, represent the core of the antioxidant system. In this review, we offer a panorama of progression of knowledge on the antioxidative system in aerobic or microaerobic (hyper)thermophilic archaea and possible industrial applications of these enzymes.

## 1. Introduction

The first forms of life evolved in an anaerobic and reducing environment, in which the main gases in the atmosphere were represented by H_2_, CH_4_, and NH_3_. Due to the photosynthetic activity of cyanobacteria, the accumulation of oxygen imposed a great selective pressure favoring the development of more complex life forms c.a. 2.2 billion years ago. The diatomic configuration of oxygen can cause the formation of reactive oxygen species (ROS) such as superoxide radical (O_2_^•−^), hydrogen peroxide (H_2_O_2_), and hydroxyl radical (HO^•^), which can damage the main biological macromolecules. As the oxygen level increased over time, microorganisms developed an effective antioxidant armamentarium to survive and protect cells from oxidative stress.

In prokaryotes, the antioxidant enzymes and the metabolism itself are intended to mitigate oxidative stress [1]. The main enzymes involved in detoxification from ROS have been widely characterized in bacteria, including superoxide dismutase (SOD), catalase (Cat), alkyl hydroperoxide reductase (Ahp), and Peroxiredoxin (Prx), in addition to enzymatic systems based on glutathione (GSH) or thioredoxin (Trx), and enzymes related to iron metabolism which are also strongly linked to oxidative stress [2,3,4,5,6,7,8].

From a metabolic perspective, the NADPH pool and the balance between the NADH and NADPH content play a key role in controlling the redox state of the cell [1]. NADPH, mainly produced by the enzymatic activities of glucose 6 P-dehydrogenase and NADP-dependent isocitrate dehydrogenase, is the source of the reducing equivalents for GSH reductase activity and the regeneration of Trx by Trx reductase (TR). Recently, the contribution of ketoacids as ROS scavengers has also been highlighted; furthermore, in bacteria the involvement of ROS-derived carboxylic acids in the protection of molecules during oxidative stress has been revealed [1].

**Figure 1:**
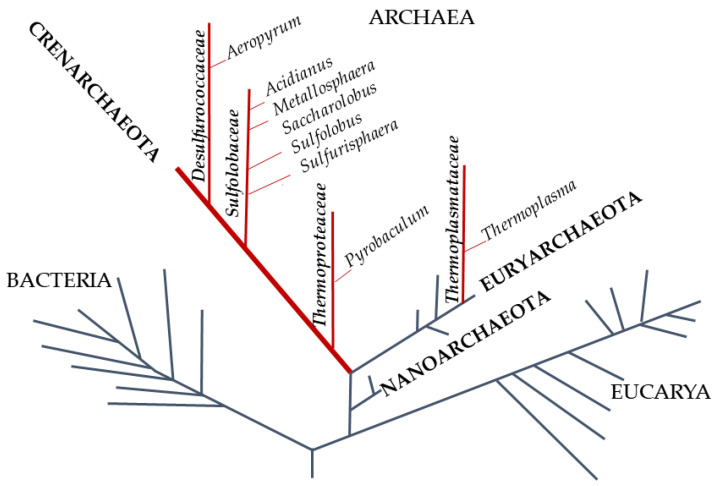
Phylogenetic tree of life. The red lines represent the hyperthermophilic aerobic archaea. In capitals, in bold italics, and in italics are phylum, family, and genus of the Archaea, respectively.

The phylogenetic tree of life, redesigned by Carl Woose in 1977, identified a third domain inside prokaryotes: The Archaea, whose genetic and biochemical characteristics are often a puzzle between Bacteria and Eucarya (Figure 1) [9].

Archaea possess many peculiar traits, such as the cell membrane composition consisting of isoprenoid lipids. Nonetheless, they appear similar to bacteria for the size, shape, DNA structure, and operon organization, and they seem more similar to eukaryotic organisms, although less complex, in processes such as transcription, translation, and cell division [10]. Archaea include microorganisms that grow in environments characterized by harsh conditions in terms of temperature, pH, salinity, and anaerobiosis; these are defined as hyperthermophiles, extreme (thermo) acidophiles, extreme halophiles, and methanogens, respectively [11].

Almost two-thirds of the archaea are hyperthermophiles [12] with an optimum growth temperature of about 80 °C. Furthermore, the majority are strict anaerobes whereas the main aerobic and microaerophilic species belong to the phylum of Crenoarchaeota. Hyperthermophilic archaea are considered the most ancestral organisms; indeed, their rDNA is deeply rooted on the phylogenetic tree of life. They are adapted to flourish in several habitats with different temperature, pH, redox potential, salinity; therefore, they represent good model systems to shed light on the evolution of enzymes and on the origin of peculiar biochemical pathways. Chemolithotrophic hyperthermophiles yield energy using H_2_ as electron source and CO_2_, Fe(OH)_3_, S^0^, SO_4_^−^, NO^3−^, and O_2_ as electron acceptor to produce methane, magnetite, hydrogen sulfite, ammonia, and water, respectively, or they gain energy using S^0^ as electron donor and O_2_ as electron acceptor. Hyperthermophiles that respire O_2_ are usually microaerophilic and therefore grow only at reduced oxygen concentrations because of the low solubility of O_2_ at high temperatures. Their terrestrial and submarine environments are mainly hot springs and sulfur-containing solfataric fields, with a wide range of pH values (pH 0–9.0), usually low salinity (0.1–0.5%), and richness in iron minerals such as ferric hydroxides and pyrite, magnetite, and arsenic minerals [13].

This review is aimed at elucidating the ancient mechanisms, which allowed the hyperthermophilic archaea to survive and adapt to oxidant environments, mainly focusing on enzymes able to scavenge ROS. We provide an overview of antioxidant enzymes identified in aerobic and microaerophilic hyperthermophiles underlining the current state of knowledge of the major players in the fight against ROS, such as SOD, Prx, and DNA-binding protein from nutrient-starved cells (Dps) and Rubrerythrin (Rbn).

## 2. Superoxide Anion Scavengers

SOD (E.C. 1.15.1.1) plays a crucial role across all life domains in removing the highly reactive O_2_^•^ through production of H_2_O_2_ and molecular oxygen.

$$2{O_{2}}^{•-}+2H^{+}\to H_{2}O_{2}+O_{2}$$

In the anaerobes SOD is functionally replaced by the superoxide reductase (SOR) (E.C.1.15.1.2) [14] which reduces, by oxidoreductase redox system [15], O_2_ to H_2_O_2_, thereby avoiding the generation of molecular oxygen [16].

$${O_{2}}^{•-}+2H^{+}+e^{-}\to H_{2}O_{2}+H^{+}$$

SODs are subdivided into four groups on the basis of their metal cofactors: Mn-SOD, Fe-SOD, Cu/Zn-SOD, and Ni-SOD [2].

**Figure 2:**
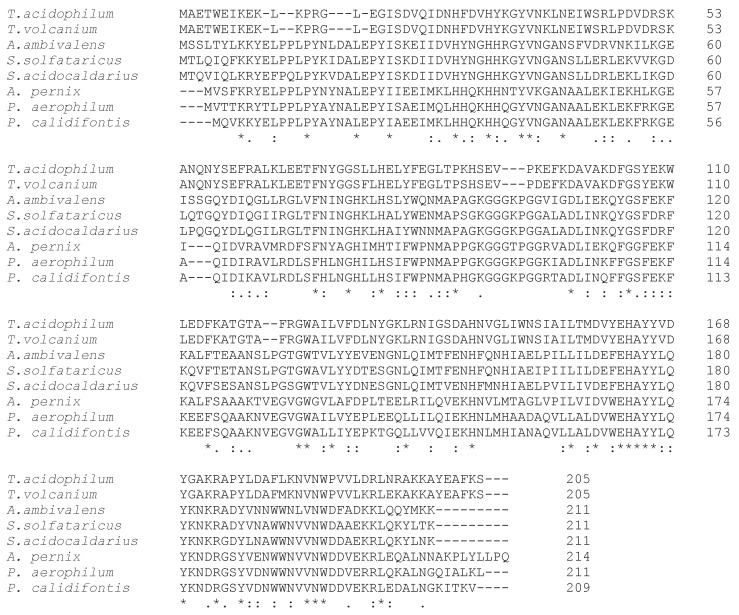
SOD sequence alignment by Clustal Omega. *Thermoplasma acidophilum, Thermoplasma volcanium, Acidianus amibivalens, Saccharolobus solfataricus, Sulfolobus acidocaldarius, Aeropyrum pernix, Pyrobaculum aerophilum*, and *Pyrobaculum calidifontis*.

Mn-SOD is common to some bacteria and archaea, and to the mitochondrial matrix; Fe–SOD occurs in other prokaryotes and chloroplasts. Particularly in bacteria, Fe- and Mn-SODs are exclusively found in the cytoplasm where they exert their action reducing membrane stress and protecting some cytosolic enzymes from inactivation by O_2_ [17]. More specifically, Fe-SOD and Mn-SOD constitute a single family showing high sequence similarity and structural homology, consisting of dimers or tetramers, thus suggesting that they originated from a common ancestor (Figure 2) [18].

Such theory is further strengthened by the discovery of specific forms of SOD named cambialistic (which means “exchange” in Latin) which can use Fe or Mn in their active site as a function of the availability of metal. [2,19]. These SODs could represent the evolutionary link missing between Fe-SODs and Mn-SODs, or could just be a novel branch created depending on metal availability.

From an evolutionary perspective, Fe-SOD and Mn-SOD could have generated in ancient forms of life when both metals were extremely bioavailable. Then, changes in the inorganic chemical makeup of the environment appear to have driven the evolution of SOD.

The ancestral versions of SOD employed Fe, consistent with the prevalence of Fe and its ready availability in the reducing environment in which life begun. However, more modern organisms utilize a version of this enzyme that requires Mn for activity, consistent with diminished bioavailability of Fe and increased Fe toxicity as O_2_ levels rose. The two enzymes show important differences in their oxidation and reduction potentials [2,19] and this feature could have contributed to the co-existence of both isoforms in different species until the present day, providing a significant advantage to organisms adapted to live in the presence of variable content of O_2_ and heavy metals.

Cu/Zn-SOD is present in eukaryotes, as both cytoplasmic and extracellular forms, and in chloroplasts; in bacteria it is found only in the periplasm of certain Gram-negative bacteria [17] and this form is totally distinct from the cytosolic SODs, not only with respect to metal content type, but also to physiological function and gene expression [17]. Cu/Zn-SOD is considered the most recent family of the SOD lineages considering that this isoform is not present in archaeal or protist genomes [2,19] and hypothesizing that it evolved much later than Fe-SOD or Mn-SOD. An additional indication of the late evolutionary origin of Cu/Zn-SOD is correlated to the increase of Cu/Zn bioavailability during or after the great oxidation event (GOE) [2,19]. Even more striking is that inside the Cu/Zn-SOD family, the extracellular form present in higher level eukaryotes looks like that of fungi more than the intracellular eukaryotic form [2,19]. This indicates either the extracellular form of Cu/Zn-SOD may be a primordial version if compared with the intracellular version which successively evolved in a divergent manner, or that both enzymes carried out the same enzymatic reactions as well as the metals used.

If compared to the amount of literature data available on the other families of SOD, really much less is known about Ni-SOD because of its recent discovery in 1996 [19,20]. Ni-SODs are primarily found in marine bacteria, *Streptomyces* sp. [21], and algae [19,22].

Differently from other SODs which function as dimers and tetramers, Ni-SOD operates as a homohexamer, whose structure does not result to be dependent upon Ni coordination [19,23]. From an evolutionary standpoint, Ni-SOD seems to be not structurally correlated to other SOD families, suggesting that it represents another example of convergent evolution for O_2_^•−^ removal. Furthermore, because it is the most predominant form in marine species, it was hypothesized that it evolved during GOE in regards to the decrease of Fe bioavailability in oceans because of increase of free O_2_ generated by oxidative photosynthesis [2,24,25].

Among the aerobic or microaerobic (hyper)thermophilic archaea, a Fe-SOD from *Saccharolobus solfataricus* (*Ss*SOD) was isolated and characterized [18]. It has a homodimeric structure and, as a result, is extremely stable. The three-dimensional structure was determined by X-ray at 2.3 Å resolution [26].

The structure revealed that *Ss*SOD forms a very compact homotetramer similar to those structurally related containing either Fe or Mn in the active site, such as SOD from the hyperthermophilic bacterium *Aquifex pyrophilus* [27]. Both structures show an elevated number of inter-subunit ion pairs compared with two other SODs, namely, that from the mesophilic *Mycobacterium tuberculosis* and that from the thermophilic *Thermus thermophilus* [26]. The crystal structure of *Ss*SOD also revealed the presence of covalent modification of Tyr41 [28], a conserved residue of Fe- and Mn-SODs located in the channel that drives O_2_ to the active site [29]. The data collected indicated that this highly reactive tyrosine residue (Tyr41) plays an important role in the enzyme activity and the maintenance of the structural architecture of *Ss*SOD. In the case of *Ss*SOD, such modification is caused by phenylmethanesulfonyl fluoride (PMSF), which reacts with the hydroxyl group of Tyr41 and causes the irreversible inactivation of the enzyme [29]. Interestingly, a similar reactivity was previously reported for the corresponding Tyr34 of human mitochondrial Mn-SOD where the modifying agent was peroxynitrite, which led to the formation of a 3-nitrotyrosine. This similarity suggests that *S. solfataricus* is a suitable model for studying the evolution of this ancient and crucial enzyme. The comparison between the thermophilic *Ss*SOD and the mammalian mitochondrial Mn-SOD could be particularly stimulating because they share different features: A compact homotetrameric organization, 38% amino acid sequence identity, a similar reactivity toward covalent modifications [28,29], and an unusual heat resistance for a mesophilic enzyme. Therefore, the *Saccharolobus* genus has been proposed as the putative ancestor of animal mitochondrial genome [26].

Interestingly, in a project aimed at the identification of released and cell-bound enzymes from *S. solfataricus*, the screening of exoproteins revealed a protein of 23 kDa as the most abundant component [17]. Primary structure investigation and specific enzyme assays identified this protein as a SOD and, surprisingly, unlike all secreted Cu/Zn-SOD found in prokaryotes, it was exactly the SOD of the Fe-type [17]. Moreover, the secreted SOD was shown to prevent deactivation by potassium superoxide of the cell-bound glucose and succinate dehydrogenases by an in vivo assay on intact cells, thus demonstrating its role in cellular protection against destructive protein oxidation. The natural habitat of *S. solfataricus* is strongly oxidative, also supporting the presence of an extracellular SOD.

Among thermophiles and hyperthermophiles, Fe-SODs were also characterized from *Sulfolobus acidocaldarius*, *Acidianus ambivalens*, and *Thermoplasma acidophilum* [30]. It is worth mentioning that homologs of all of the *S. solfataricus* oxidative stress response elements were identified in the *Metallosphera sedula* genome and several of these were induced by metal challenge; among these, the presence of a SOD (Msed_1889) must be highlighted [31].

Mn-SODs have been also described in thermophilic bacteria such as *T. thermophilus* and *Chaetomium thermophilum*. Moreover, cambialistic SODs, which are active with either Mn or Fe, have been described in *Pyrobaculum calidifontis* or *P. aerophilum* [30,32], or *Aeropyrum pernix* [33] (Table 1). To date, Cu/Zn-SODs have not been found in archaea.

Interestingly, the archaea that harbor SOD do not necessarily rely upon a catalase. An exception is represented by *P. calidifontis*, which is the only hyperthermophilic facultative aerobe archaeon known to utilize a SOD-catalase system to detoxify ROS [30].

## 3. H_2_O_2_ Production

H_2_O_2_ can be produced exogenously by chemical processes or by the action of competing organisms and enters cells through the membrane because it has the same permeability coefficient of water [34]. In general, H_2_O_2_ and ROS can cause an altered protein structure favoring aggregation, protein–protein cross-linking, hydrophobic bonds, and increasing susceptibility to proteolysis, and affect the normal cellular functions [35]. At the same time, H_2_O_2_ can be formed endogenously by SOD and SOR activities as reported above, by autoxidation of flavoproteins [36]. Furthermore, NADH oxidase (NOX) can be involved in H_2_O_2_ production and detoxification [37,38] inside the cell. NOXs are found in several microorganisms belonging both to Bacteria and Archaea. They are members of the Flavinprotein disulfide reductase family and contain two highly conserved domains, one binding Flavin Adenine Nucleotide and the other NAD(P)H. NOXs can catalyze the reduction of several substrates such as O_2_, H_2_O_2_, and Trx. They play an important role in the regeneration of NAD in the aerobic metabolism but can also be involved in the removal of peroxides [38]. Generally, there are two types of NOXs: One catalyzes the 2-electron reduction of O_2_ to H_2_O_2_ by NAD(P)H and the other catalyzes the 4-electron reduction of O_2_ of H_2_O by NAD(P)H [39,40]. NOX functions are still enigmatic and show different physiological roles in different microorganisms. In the anaerobic archaeon *Archeoglobus fulgidus*, eight putative NOXs were revealed from genome analysis, among which NOX A-2 is involved in electron transport during sulfate respiration [41], in the facultatively anaerobic bacterium *Amphibacillus xylanus*. NOX is a part of the alkyl hydroperoxide reductase system to detoxify H_2_O_2_ [42]; in the anaerobic archaeon *Pyrocccus furiosus* NOX-1 produces both H_2_O_2_ (77%) and H_2_O (23%) [43,44]; and in *Thermococcus profundus* NOX catalyzes the electron transfer from NAD(P)H to O_2_ producing mainly H_2_O [39]. NOXs in anaerobic thermophilic microorganisms probably play a key role in O_2_ removal to allow aerobic tolerance [39]. In aerobic *S. solfataricus* a homodimeric NOX (*Ss*NOX38) has the main role in the regeneration of NAD from NADH produced in the aerobic pathway and it does not show peroxidase activity [45]. In fact, differently to other NOXs whose peroxidase activity is due to a single highly conserved redox active cysteine residue [43,44], *Ss*NOX38 lacks cysteine residue in its primary structure. The significant homology of *Ss*Nox38 with TR and alkyhydroperoxide reductase of *Bacillus subtilis* and *Xhantomonas campestris*, respectively, is found in the region containing the binding sites for FAD and NAD(P), and the main divergence is represented by the lack of a redox center, supporting the hypothesis that the enzyme does not possess reductase activity [40].

## 4. H_2_O_2_ Targets

The main targets of H_2_O_2_ are thiol groups of cysteine residues that, in proximity to positively charged residues, can be deprotonated to thiolate anions (-S-), potent nucleophiles that promptly react with ROS, forming sulfenic (-SOH), sulfinic (-SO_2_H), and sulfonic (-SO_3_H) acids. The reactivity of the thiol group with H_2_O_2_ is related to its accessibility and the repair of oxidized Cys residues is due to different ubiquitous redox systems including TR/Trx and Glutaredoxin (Grx)/GSH/GSH reductase [8,46].

H_2_O_2_ can also determine the oxidation of the sulfur atom in the side chain of methionine residues with the consequent generation of diasteroisomers of methionine sulfoxide and successively of methionine sulfone [47]. Oxidation of methionine by ROS can lead to carbonylation, aggregation, and degradation of proteins. The methionine sulfone oxidation state is irreversible, while methionine sulfoxide can be repaired to methionine by Methionine Sulfoxide Reductases (MSRs). These enzymes are grouped into two distinct families with different structures and substrate specificities sharing a common catalytic mechanism in which a cysteine nucleophile and a resolving cysteine or a single cysteine in the active site are able to reduce methionine sulfoxide [48]. The oxidized enzyme is generally regenerated by disulfide redox systems [49]. Another H_2_O_2_ target is the Fe-S proteins which are inactivated due to the release of Fe^2+^ that, in turn, favors other ROS formation [36].

To defend from H_2_O_2_ attack, an array of enzymes is available in cellular systems, such as catalases and several type of peroxidases; the reason for this abundance is not only to preserve the biomolecules from oxidative damage but also to prevent the formation of HO. The generation of this radical, produced by the Fenton reaction, is related both to H_2_O_2_ and to the pool of Fe^2+^; this ion is indispensable not only for antioxidant enzymes such as Fe-SOD and heme catalase, but also in pathways needed for the reduction of ribonucleotides and electron transfer [50].

(1) $$\mathrm{Fenton}\;\mathrm{reaction}\;H_{2}O_{2}+{\mathrm{Fe}}^{2+}\to {\mathrm{OH}}^{-}+{\mathrm{OH}}^{•}+{\mathrm{Fe}}^{3+}$$

For this reason, the H_2_O_2_ and Fe^2+^ concentration inside the cell is strictly controlled as described in more detail below [36].

## 5. The Main Peroxide Scavengers in Hyperthermophilic Aerobic/Microaerophilic Archaea: Prxs

The characterization of scavenging H_2_O_2_ enzymes in hyperthermophilic archaea is in its infancy in comparison to bacteria; heme catalase was characterized in anaerobic methanogenic archaea as *Methanosarcina barkeri* [51] and *Methanobrevibacter arboriphilus* [52], in halophilic archaea as *Halobacterium halobium* [53], and in obligate anaerobic hyperthemophilic archaea as *Archaeglobus fulgidus* [54]. However, genome sequences from aerobic (hyper)thermophiles such as *S. solfataricus* [55], *Sulfurisphaera tokodaii* [56], *A. pernix* [57], *T. acidophilum* [58], and *T. volcanium* [59] did not show catalase orthologues with an exception in *P. aerophilum* [60,61] and *P. calidifontis* [62], which are facultative aerobic archaea whose genomes encode catalases (Cats). In particular, these genes are transcriptionally induced by the presence of oxygen and the Cat of *P. calidifontis* is the first example in archaea of a characterized Mn catalase [62].

Based on the most recent published results, the majority of aerobic hyperthermophilic archaea use Prxs to eliminate peroxides; thus, in this review we focus on this class of enzymes.

**Figure 3:**
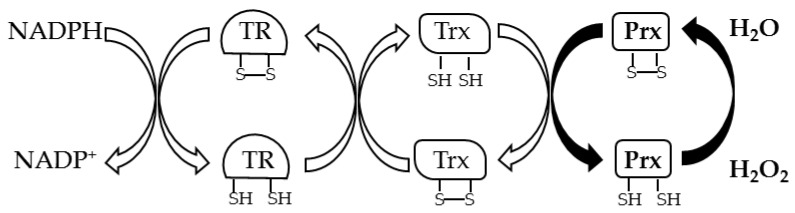
General thiol redox pathway to recycle Prxs.

Prxs (EC 1.11.1.15) are thiol peroxidases that are characterized by a Trx fold in which one or two cysteine residues play a key role in the reduction of inorganic and organic peroxides [5]. These enzymes are classified in 1-Cys Prxs and 2-Cys Prxs based on the number of cysteine residues involved in the catalysis. 1-Cys Prxs have only one Cys residue located at the N-terminus of the protein, the peroxidatic cysteine (C_P_), which is oxidized to sulfenic acid in the presence of peroxide. The regeneration of the enzyme in the reduced active state is generally due to GSH or other disulfide reducing systems. 2-Cys Prxs can be divided into atypical 2-Cys Prxs and typical 2-Cys Prxs based respectively on the formation of an intramolecular or intermolecular disulfide bond between C_P_ and the resolving cysteine (C_R_), which is the other catalytic residue. In atypical 2-Cys Prxs, the C_R_ is located at the N-terminus, 10–15 aa from C_P_, or at the C-terminus. In both cases an intramolecular disulfide bond is formed after the oxidation of C_P_. In contrast, in the typical 2-Cys Prxs, the C_P_ at the N-terminus and C_R_ at the C-terminus belong to different subunits and condense to form an inter-subunit disulfide bond; in fact, the 2-Cys Prxs have a complex quaternary structure as the hexadecameric structure described in *A. pernix* [63]. In both cases a disulfide reductase system recycles Prxs; generally, an electron cascade starting from NADPH through TR and Trx reduces the disulfide bond of Prx (Figure 3).

### 5.1. Prxs in Sulfolobaceae Family

Prxs are assigned into six distinct subfamilies (AhpC/Prx1, Prx6, Bcp/PrxQ, Prx5, Tpx, or AhpE) based on the PeroxiRedoxin classification index (PREX) (http://www.csb.wfu.edu/prex/) which uses the Deacon Active Site Profiler (DASP) to highlight functionally relevant sequences that surround key residues required for peroxidase activity [64]. Archaeal Prxs belong to Bcp/PrxQ or Prx6 subfamilies [64]; inside the Crenoarchaeota phylum, the *Sulfolobaceae* family includes the genera *Saccharolobus*, *Sulfolobus*, *Sulfurisphaera*, and *Metallosphaera* in which several species possess Prxs. The genome of *S. solfataricus* [55] does not contain genes encoding putative catalase, glutathione peroxidase, or alkyl hydroperoxide reductase; on the contrary, it possesses four Prxs: Bcp1, Bcp2, Bcp3, and Bcp4 [65]. Bcp2 is a 1-Cys Prx of the Prx6 subfamily with only one conserved cysteine residue (Cys49) in a consensus surrounding the sequence DFTPVCTTE conserved both in prokaryotic and eukaryotic Prxs [66,67], while Bcp1, Bcp3, and Bcp4 were classified as belonging to the Bcp/PrxQ subfamily [65]. Bcp2 shows lower sequence identity and has a different molecular mass (~24 kDa) in comparison to Bcp1,Bcp3, Bcp4 (~17 kDa). Bcp2 shares 95% of sequence identity with 1-Cys Prx from *S. islandicus* (*Si*Pr), 61% with the putative bacterial Prx (Q9WZR4) from the hyperthermophilic bacterium *Thermotoga maritima*, and even 40% identity with 1-Cys Human Prx6. Transcriptional analysis of *bcp2* revealed that the specific mRNA transcript is induced under oxidative stress. Bcp2 was expressed in Escherichia coli and the recombinant protein showed maximum activity between 80 and 90 °C and a strong thermoresistance [66]. Size-exclusion chromatography showed that the protein can shift from a monomer to a multimeric form in a protein concentration- and temperature-dependent manner; the formation of a quaternary structure could be correlated to an intrinsic chaperone activity as also observed for other archaeal 1-Cys Prxs able to protect proteins and DNA from damage caused by oxidative and thermal stresses [66,68]. Recently the structure of a 1-Cys Prx of *S. islandicus* (*Si*Prx), belonging to the Prx6 subfamily was resolved [67]. In the crystal structure, *Si*Prx assembles in a decamer composed of five homodimers. In addition, in solution the predominant decameric form with respect to the dimeric organization suggests an additional activity as a chaperone [67]. Table 2 reports Prxs identified in the *Sulfolobaceae* family. Furthermore, Bcp1, Bcp3, and Bcp4 showed high thermophilicity and thermoresistance [65].

Bcp1 and Bcp4 3D structures determined by X-ray crystallography [69,70] showed that a monomeric organization for Bcp1 and a dimeric structure for Bcp4 is probably responsible for its higher thermostability. All of the Bcps of *S. solfataricus* are able to protect nucleic acids from oxidative damage and, in particular, Bcp3 and Bcp2 are more efficient than Bcp1 and Bcp4 to ensure DNA integrity. Furthermore, the rapid bcp2 and bcp3 mRNA increased when the cells were grown in the presence of peroxides, suggesting that Bcp2 and Bcp3 can play a primary role in response to oxidative stress, while Bcp1 and Bcp4, constitutively expressed, could be involved in the protection of cells from endogenous peroxides formed during metabolism.

Interestingly, the regeneration system of Bcp1, Bcp3, and Bcp4 highlighted a novel disulfide reductase system in which the reducing equivalents from NADPH and Tr are transferred to the Protein Disulfide Oxidoreductase (PDO) instead of Trx [65,71,72]. PDOs have been exclusively found in thermophiles and are characterized by a high disulfide content of cytoplasmic proteins [24,73,74,75], suggesting for them a crucial role in the adaptation to conditions of extreme temperature. PDOs have a molecular mass of 26 kDa, and are organized into two Trx folds (a four-stranded central β-sheet and three flanking α-helices), each with a CXXC active-site motif, one at the N-terminus and the other at the C-terminus [72]. Mutagenesis studies performed on *S. solfataricus* PDO (SsPDO) have clarified the contribution of each active site to the overall catalytic activity, showing that the C-terminal site has a fundamental role in the thiol-transferase activity; both sites are indispensable for isomerase activity, and the two Trx folds are presumed to function synergistically. An ATP dependent chaperone activity was determined for SsPDO [76], as also reported for the eukaryotic Protein Disulfide Isomerase (PDI) [77], further supporting the correlation between PDO and PDI.

Homologs of *S. solfataricus* Bcp4 and PDO were also identified in the genome of *M. sedula* (Msed_0359 and Msed_0153, respectively) [31]. In particular, the expression of the Bcp4 homologs was induced upon exposure to high doses of Co^2+^, Cu^2+^, and Zn^2+^ revealing a relationship between oxidative stress and metal shock.

### 5.2. Prxs in the Desulfurococcaceae Family

In the Crenoarchaeota phylum, inside the *Desulfurococcaceae* family, *Aeropyrum pernix* is an aerobic hyperthermophile, whose 1-Cys Prx (*Ap*Tpx) was characterized [78]. This Prx, which belongs to the Prx6 subfamily, shares 61% of its identity with *Si*Prx of *S. islandicus* and Bcp2 of *S. solfataricus* [66]. In contrast to *Si*Prx, however, it is assembled in a hexadecameric structure organized in a two-fold toroid-shaped particle [79]. The structure is not maintained by disufide bonds as it is not destroyed in the presence of dithiothreitol; in contrast, the octamers are dissociated into monomers under reducing conditions underlining intersubunit disulfide bonds. Site-directed mutagenesis on Cys50, Cys207, and Cys213 indicated that each of these three residues is important for peroxidase activity, and Cys50 corresponds to C_P_. In the proposed mechanism, the enzyme is regenerated by the NADPH/TR/Trx reducing system, which reduces the disulfide bond between Cys50 and Cys213 of *Ap*Tpx.

*Ap*Tpx orthologs were also found in anaerobic hyperthermophilic members of the Euryarchaeota phylum such as *P. horikoshii* (*Ph*Prx) and *Thermococcus kodakarensis* (TkPrx), which share 58.7% and 55% sequence identity, respectively. *Tk*Prx displays different oligomeric structures; in particular, the dimer shows peroxidase activity, while dodecamers own chaperon and DNA binding activities able to prevent protein aggregation and DNA damage during oxidative stress. Similarly to Prxs of *T. kodakariensis* and *S. islandicus,* the additional role of chaperon related to its quaternary structure could also be ascribed to *Ap*Trx [67].

## 6. Other Enzymes Involved in the Cellular Protection against Oxidative Stress

### 6.1. DNA-Binding Protein from Starved Cells (Dps)

Although the level of peroxides is controlled by an array of peroxidases and catalases, that of Fe^2+^ must also be limited to prevent the Fenton reaction. The main strategy is based on the mineralization of the free ferrous ions by proteins belonging to the Ferritin like superfamily, such as Ferritin (Ftn), Bacterioferritin (Bfr), Dps, and, recently, Dps-like (Dpsl) proteins. All of these proteins are composed of at least 12 subunits folded into four-helix bundles with a di-metal binding site (ferritin domain). In Ferritin/Bacterioferritin the iron binding site is located within the four-helix bundles, while in Dps it is found at the interface between the two subunits. The members belonging to this superfamily share a common ancestor and are assembled into cage-like structures [80,81].

Dps, initially discovered in *E. coli* cells during the stationary phase, is ubiquitously present in the bacterial and archaeal kingdoms, and is involved in H_2_O_2_ detoxification, iron scavenging to prevent Fenton reaction, and DNA protection [82,83,84]. Differently from Ftn, Dps can store up to 500 iron atoms in the hollow cavity in comparison to 3000 iron atoms per protein molecule accumulated in Ftn. In contrast to Ftn and Btr, which use oxygen, Dps catalyzes the oxidative conversion of Fe^2+^ to Fe^3+^ in the presence of H_2_O_2_, using ferroxidase centers (FOCs) as reported in the following reaction.

(2) $${\mathrm{Fe}}^{2+}+H_{2}O_{2}+H_{2}O\to 2{\mathrm{Fe}}^{3+}\;\mathrm{OOH}(\mathrm{FOC})+4H^{+}$$

Generally, FOCs consist of two Fe^2+^ cations that are coordinated by highly conserved amino acids, including two His, one Trp, one Asp, and one Glu [85]. Dpsl proteins display a common three-dimensional architecture characterized by spherical dodecamers with a hollow central cavity for iron storage. Over one thousand Dpsl proteins have been identified (http://www.uniprot.org) but only a low percentage were found in archaea. In addition to protection from oxidative stress and DNA-binding, Dpsl proteins possess several activities that protect against multiple stresses, including metal stress, om addition to heat and cold shock [86,87,88].

Dpsl was characterized in *S. solfataricus* (*Ss*Dpsl) [89] and in the anaerobic archaeon *P. furiosus* [80]. *Ss*Dpsl has a dodecameric structure characterized by three lysine residues protruding at the N-terminal which adopts an α-helical conformation that can interact with the DNA major groove. Similarly to other Dpsl, the proximal couple of cysteine residues (Cys101 and Cys126) is collocated between the exterior surface of the structure and the channel that connects the FOC, and can form a disulfide bond. A thio–ferritin motif is typical of the Dpsl protein together with the ferroxidase-like active site.

*Ss*Dpsl catalyzes the consumption of H_2_O_2_ and Fe^2+^, preventing HO^•^ and the expression of the corresponding gene (*Sso*2079) is over-expressed in response to H_2_O_2_ [89,90] together with a hypothetical neighbor protein (*Sso*2078) probably involved in the Fe^2+^ transport. Transcriptomic and proteomic analyses also highlighted the increased expression of oxidative stress-related proteins such as SOD and Bcp. Moreover, size exclusion chromatography performed using H_2_O_2_-stressed cells suggested that *Ss*Dpsl could be part of a large molecular complex in which Bcp and SOD could be the interactors [90] to fight Fe^2+^, H_2_O_2_, and O_2_^•^ respectively.

### 6.2. Rubrerythrin (Rbr)

Among the H_2_O_2_-scavenging enzymes, Rbr is a non-heme di-iron protein also belonging to the Ferritin like superfamily, with an important role in stress survival in several microorganisms [81,91].

**Figure 4:**
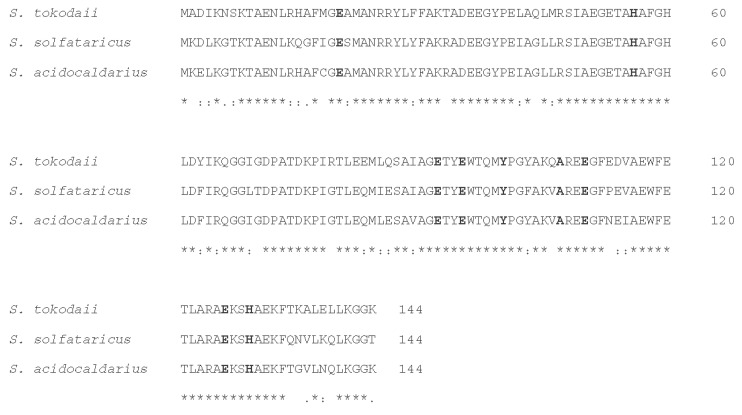
Sequence alignment of Sulerythrin by Clustal Omega among *S. tokodai, S. solfataricus,* and *S. acidocaldarius*. Conserved residues that are related to di-iron center binding are in bold.

Rbr, together with SOR, is found predominantly in air-sensitive bacteria and archaea, and functions as a scavenger of ROS [92]. In Rbr, the di-iron center is responsible for the reduction of H_2_O_2_ and organic hydroperoxide [81,93]. The role of Rbr in the reduction of H_2_O_2_ has been experimentally verified in several different organisms including aerobes [81,94], cyanobacteria [81,95], and obligate anaerobes [81,96]. The classic Rbr, found in the obligate anaerobe *Desulfovibrio vulgaris*, contains, as revealed by crystallographic structure [81,97], in addition to the ferritin domain at the N-terminus (residues 1–145), a C-terminal domain (residues 150–195) related to the Rubredoxin family, putatively involved in the electron transfer during catalysis [81]. This feature is conserved in most representatives of this family [98], including “reverse Rbr”, which is a version of Rbr with its rubredoxin domain in the opposite orientation with respect to the classic version [92], suggesting that the rubredoxin-like domain has an important role in Rbr function. Despite the strong conservation of the rubredoxin-like domain in Rbr proteins, there are also versions of this enzyme without the C-terminal rubredoxin-like domain such as the Sulerythrin found in *S. tokodaii* [98]. This is the first member of a Rbr family discovered in aerobic archaea in the *Sulfolobaceae* family with the smallest size known to date (135–144 amino acid residues) (Figure 4). The physiological function of Sulerythrin is not clear, although it may play a role in oxygen binding or in defense against oxidative stress.

The phylogenomic study reported by Cardenas et al. [91] proposes the means by which Rbrs could have evolved: A complete lineage of Rbrs, lacking the rubredoxin-like domain, arose in microaerobic and (hyper)thermophilic environments in which the ancestors of *Thermoproteales* and *Sulfolobales* lived [91]. This aerobic-type lineage subsequently evolved to become adapted to environments with progressively lower temperatures and higher oxygen concentrations via the acquisition of two co-localized genes, termed DUF3501 and RFO, encoding a conserved protein of unknown function and a predicted Fe-S oxidoreductase, respectively. Proposed Horizontal Gene Transfer events from these archaeal ancestors to Bacteria expanded the opportunities for further evolution of this Rbr including adaption to lower temperatures.

## 7. Biotechnological Applications

Knowledge of oxidative stress resistance mechanisms in hyperthermophilic archaea is receiving more attention, not only from an evolutionary point of view, but also from a biotechnological perspective in different fields such as environment, agriculture, industry, pharmacology, therapeutics.

However, despite the great number of antioxidant enzymes/proteins from hyperthermophilic Archaea that have been biochemically characterized for their applicative potential, only a few have been applied in biotechnology.

A detailed understanding of the molecular mechanisms responsible for the response to oxidative stress is fundamental for engineering organisms and developing bioreporters to measure the bioavailability of many emerging pollutants in the environment. In this regard, bioreporter strains that respond to oxidative stress can be constructed using the promoters of the genes encoding antioxidant enzymes, such as Prx or SOD, which are able to respond to increased oxidative stress, fused to suitable reporter genes [99]. Although bioreporter strains have not yet been developed for hyperthermophilic archaea, the utilization of thermophilic molecular components and thermostable chassis cells has already been considered promising in biosensing applications [100]. Moreover, Prxs have also been considered a highly useful tool in applied research as components of biosensors for the real-time observation of H_2_O_2_ in living cells. An increasing number of studies highlights either the roles of Prxs in different diseases, such as diabetes, neurological disorders, and cardiovascular diseases, or the need to investigate their use as antioxidants or anti-inflammatories in living systems [101,102].

Genes encoding H_2_O_2_ fluorescent sensors consist of (at least) two components; the first is a fluorescent reporter protein, and the second is a Prx representing the sensitive protein domain that can undergo a conformational change in response to H_2_O_2_ [103]. Recently, a novel Prx-based sensor was realized using a bacterial Prx able to detect H_2_O_2_ with high sensitivity [104]. It can be speculated that Prxs from hyperthermophilic archaea can be even more successfully used as moieties for the development of rapid and sensitive H_2_O_2_ sensors in living cells thanks to their higher stability in comparison to mesophilic counterparts. The development of Prx-based probes may in turn encourage further studies of H_2_O_2_ homeostasis and Prx function in the Archaea.

New insights into the possibility of using archaeal sources of antioxidant enzymes, such as those from thermophilic microorganisms, were previously reported by our research group; Sarcinelli et al. [102] demonstrated that Bcp1, an archaeal Prx from *S. solfataricus*, functions as an antioxidant in eukaryotic cells; in H9c2 rat cardiomyoblasts, the Prx is not cytotoxic and is able to both reduce the endogenous peroxide levels and decrease cellular apoptosis following H_2_O_2_-induced stress [102]. Taking advantage of its exceptional stability and function, Bcp1 was also efficiently and stably adsorbed onto spores of *Bacillus megaterium* and was demonstrated to maintain antioxidant activity even in simulated intestinal or gastric conditions. Hence, the spore-based system can be considered a promising new tool to address inflammation caused by oxidative stress as it represents an oral delivery vehicle able to transport the beneficial antioxidant molecule at the level of the intestinal mucosal surfaces [105].

Antioxidant enzymes have also been deeply studied and overexpressed since they are used in several industrial applications; in this context, SODs are widely used in cosmetics, health care products, because they reduce free radical damage and promote general health of the body; SOD is in fact a common constituent of several cosmetic creams as well as dietary supplements [106]. Despite the considerable research and the number of SOD-related inventions and patents done, SOD has not yet received approval for human therapy [106]. Nevertheless, all the clinical research work has only focused on human and bovine SOD whereas SODs from alternative sources still hold promises for the future. In this respect, hyperthermophilic SOD able to work under different conditions may provide greater versatility in therapeutics.

In a recent work, SOD was loaded into nanoarchaeosomes that are nanovesicles made of archaeolipids to protect and target SOD to inflammatory macrophages upon oral administration: It was shown that nanoarchaeosomes retained higher antioxidant and anti-inflammatory activity in comparison to SOD encapsulated into highly stable liposomes [107].

The thermostability associated with a higher tolerance to chemical denaturants of the enzymes from hyperthermophiles makes them attractive for industrial applications requiring harsh conditions, such as high temperature, strong acid and alkali, or organic and denaturing media [108].

Most native SODs characterized from hyperthermophilic archaea have been analyzed for industrial applications and, in some cases, site-directed mutagenesis has been used for improving their thermostability as reported for Fe-SOD from *A. pyrophilus* [109]. Recently, a general and feasible strategy has also been proposed to enhance the thermophilicity and tolerance of both SODs from either bacteria or archaea [110]. The proof of principle description was based on the genetic fusion of SOD from *S. solfataricus* with the N-terminal domain of SOD from *Geobacillus thermodenitrificans*. The resulting enzyme showed enhanced thermostability, greater stress resistance and tolerance to organic solvents, and stability over a wider pH range.

The overexpression of thermophilic antioxidant enzymes has also been exploited as application in the agriculture as well as for increasing the thermotolerance of economically valuable insects; heat shock, such as variations in the environmental temperature, can determine harmful production of ROS in organisms employed in sericulture, resulting in dramatic economic losses. In this context, discovery and application of genes promoting high temperature tolerance and/or antioxidant capability are essential to enhance crop tolerance to heat stress.

This problem was solved by the generation of a silkworm strain with high heat tolerance which overexpresses an archaeal gene encoding for the SOR [16]. This finding provides a strategy for increasing the thermotolerance of insects or even plants so that their cultivation can be expanded to geographical regions with temperatures higher than optimal [111]. On the other hand, a PDI from *Methanothermobacter thermautotrophicus* delta H, was ectopically expressed in rice and demonstrated to be able to confer heat stress tolerance of transgenic rice and a synergistic increase of the antioxidant enzymes activity with increased production of thiols and relief from the oxidative damage [112].

In recent times, extremely thermophilic archaea have also been exploited as new platforms for performing metabolic engineering at high temperatures. This field either provides new opportunities for the commercial application of thermo-enzymes or can be considered a source of novel biosynthetic pathways for producing bio-based fuels and chemicals [113]. In this regard, some studies have reported means to improve microbial tolerance to oxidative and temperature stress; such achievements can be used as the starting point to further engineer selected strains and improve their metabolic robustness.

## 8. Conclusions

**Figure 5:**
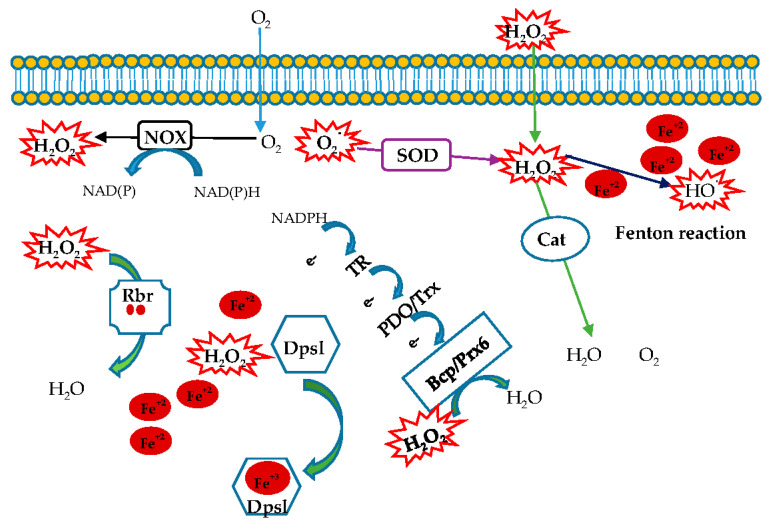
Schematic response of the enzymes involved in oxidative stress in hyperthermophilic aerobic archaea.

An enriched literature regarding oxidative stress in eukaryotes and bacteria is reported, while this topic in archaea is still unexplored. In this review we focused on antioxidant enzymes from hyperthemophilic aerobic archaea acting as ROS scavengers (Figure 5). This study can help to understand both how these microorganisms have adapted to fight ROS in extreme environments and how such an armamentarium of enzymes has evolved. Furthermore, the peculiar features of these enzymes reveal a new biotechnological scenario involving robust tools to be used in industrial processes.

## Figures and Tables

**Table 1:** SODs in aerobic or microaerobic hyperthermophilic archaea.

Fe-SOD	Cambialistic SOD
*Saccharolobus solfataricus*	*Pyrobaculum aerophilum*
*Sulfolobus acidocaldarius*	*P. calidifontis*
*Acidianus ambivalens*	*Aeropyrum pernix*
*Thermoplasma acidophilum*	

**Table 2:** Genes encoding Prxs in the *Sulfolobaceae* family. In brackets are the characterized proteins.

*Saccharolobus solfataricus*	*Sulfolobus islandicus*	*Sulfolobus acidicaldarius*	*Sulfurisphaera tokodaii*
SSO_RS10090 (Bcp1) [69]	SIRE_RS03670	SACI_RS10765	STK_RS04035
SSO_RS10350 (Bcp2) [65,66]	SIRE_RS01755(*Si*Pr) [67]	-	STK_RS13435
SSO_RS11005(Bcp3) [65]	SIRE_RS00330	SACI_RS00260	STK_RS11650
SSO_RS12680 (Bcp4) [70]	SIRE_RS13090	SACI_RS05365	STK_RS09985

## Abbreviations

Superoxide dismutase (SOD), catalase (Cat), NADH oxidase (NOX), peroxiredoxin (Prx), Bacterioferritin comigratory protein (Bcp), Thioredoxin (Trx), Thioredoxin reductase (TR), DNA-binding Protein from Starved cells (Dps), Ferritin (Ftn), Bacterioferritin (Bfr), Rubrerythrin (Rbr).
